# Regulatory Information and Guidance on Biosimilars and Their Use Across Europe: A Call for Strengthened One Voice Messaging

**DOI:** 10.3389/fmed.2022.820755

**Published:** 2022-03-09

**Authors:** Liese Barbier, Allary Mbuaki, Steven Simoens, Paul Declerck, Arnold G. Vulto, Isabelle Huys

**Affiliations:** ^1^Department of Pharmaceutical and Pharmacological Sciences, KU Leuven, Leuven, Belgium; ^2^Hospital Pharmacy, Erasmus University Medical Center, Rotterdam, Netherlands

**Keywords:** regulatory, biosimilar, biological, guidance, switching, interchangeability, substitution, policy

## Abstract

**Background:**

Beyond evaluation and approval, European and national regulators have a key role in providing reliable information on biosimilars and the science underpinning their development, approval, and use.

**Objectives:**

This study aims to (i) review biosimilar information and guidance provided by EMA and national medicines agencies and (ii) explore stakeholder perspectives on the role of regulators in enabling acceptance and use of biosimilars.

**Methods:**

This study consists of (i) a comparative review of regulatory information and position statements across medicine agencies (*n* = 32) and (ii) qualitative interviews with stakeholders in Europe (*n* = 14).

**Results:**

The comparative analysis showed that regulatory information and guidance about biosimilars offered by national medicines agencies in Europe varies, and is limited or absent in multiple instances. Approximately 40% (13/31) of the national medicines agencies' websites did not offer any information regarding biosimilars, and for about half (15/31) no educational materials were provided. Only less than half of national medicines agencies provided guidance on biosimilar interchangeability and switching (8/31 and 12/31, respectively). Among the national medicines agencies that did offer guidance, the extent (e.g., elaborate position vs. brief statement) and content (e.g., full endorsement vs. more cautious) of the guidance differed substantially. Countries that have a strong involvement in EU level biosimilar regulatory activities generally had more elaborate information nationally. Interviewees underwrote the need for (national) regulators to intensify biosimilar stakeholder guidance, especially in terms of providing clear positions regarding biosimilar interchangeability and switching, which in turn can be disseminated by the relevant professional societies more locally.

**Conclusion:**

This study revealed that, despite strong EU-level regulatory biosimilar guidance, guidance about biosimilars, and their use differs considerably across Member States. This heterogeneity, together with the absence of a clear EU-wide position on interchangeability, may instill uncertainty among stakeholders about the appropriate use of biosimilars in practice. Regulators should strive for a clear and common EU scientific position on biosimilar interchangeability to bridge this gap and unambiguously inform policy makers, healthcare professionals, and patients. Furthermore, there is a clear opportunity to expand information at the national level, and leverage EU-developed information materials more actively in this regard.

## Introduction

With the expiration of patents and other exclusivity rights on many best-selling and high-cost biologics, biosimilar alternatives have gradually been entering the European market over past years. As defined by the European Medicines Agency (EMA), a biosimilar is a biological medicine that is highly similar in quality, safety, and efficacy compared to an already approved biological product (also called the reference product) (1, 2). Biosimilar market entry and the resulting price competition has shown to positively impact healthcare systems across Europe, in terms of lowering treatment cost of biological therapies and in some instances by broadening patient access to biological medicines (3, 4). Europe has pioneered the regulation of biosimilars by establishing a robust regulatory framework for marketing authorization in 2004, and the very first biosimilar approval (Omnitrope®, a biosimilar of somatropin) in 2006 (1, 5).

Over the past 15 years, considerable experience with biosimilar evaluation has been accumulated, and the EMA has issued and updated scientific guidelines outlining biosimilar development data requirements (6). Biosimilar approval is based on the demonstration of biosimilarity, i.e., a high level of similarity to the reference product in terms of quality, safety, and efficacy to the reference product. To this end, comprehensive comparability studies with the reference product are carried out (1, 2). With the exception of some low-molecular weight heparins, all biosimilars approved for use in the EU have been approved via the centralized procedure, i.e., through the EMA, as they use biotechnology for their production (1). Since the first biosimilar approval in 2006, over 65 biosimilars have been granted marketing authorization in Europe, and are available in different disease areas such as endocrinology, hematology, rheumatology, gastroenterology, and oncology (7). The European biosimilar landscape is likely to continue to expand in future years. Presently, 10 biosimilar marketing authorization applications are under review by EMA's Committee for Medicinal Products for Human Use (CHMP) and ~120 originator biologicals products are expected to lose exclusivity in the next 10 years, opening up more opportunities for biosimilar development and competition (8).

Despite the strong EU track-record in terms of biosimilar evaluation and approval, which resulted in the availability of a multitude of biosimilar products with an EU-wide marketing authorization, biosimilar adoption has been of varying success across healthcare systems and products (4). Reasons for low biosimilar use are multifaceted and some may be specific to local context and healthcare organization. However, overall, one of the main commonalities appears to be a limited understanding of biosimilars among healthcare providers and patients which in turn may hamper willingness to use them (9). Several studies have shown rather limited knowledge and confidence levels in biosimilars among European healthcare providers and patients, indicating uncertainty and resulting in hesitation to use them (10–18). Limited understanding and trust in biosimilars may in part be explained by the fact that the science underpinning biosimilar development poses a new paradigm, different from that of the development of novel drugs, for stakeholders to become acquainted to, understand and trust, and a general lack of understanding of biological medicines and biotechnology (19, 20). Furthermore, disparagement and misinformation about biosimilars, whether intentional or otherwise, is considered to have strongly contributed to misconceptions about biosimilars among healthcare providers and patients (21, 22).

Over the past years, the science behind biosimilars has been progressively adopted by healthcare professional societies, endorsing biosimilar use in their position statements (23–25). The EMA, together with the European Commission (EC), took an active stance and made considerable efforts in developing biosimilar educational resources for healthcare professionals and patients. The EC committed itself to the organization of a yearly multi-stakeholder conference on biosimilar medicines, providing a platform to relevant stakeholders to share experiences on the use of biosimilars and discuss relevant policy choices and practices (26). Also national medicines agencies, and various healthcare professional and patient organizations on both pan-EU and national level did so (1, 26–31). Yet, uncertainties and a general lack of familiarity with biosimilars appear to persist among the broader population of healthcare professionals and patients, underlining the need for continued information and guidance and possibly more integrated approaches in terms of reaching the relevant stakeholders (9, 32).

Guidance may be especially needed regarding the interchangeable use of biosimilars with their reference product, since most best-selling biologicals are used in a chronic setting (33). Interchangeability is defined as “*the possibility of exchanging one medicine for another medicine that is expected to have the same clinical effect. This could mean replacing a reference product with a biosimilar (or vice versa) or replacing one biosimilar with another*” (1). Questions on the appropriateness of exchanging a reference product with a biosimilar (or *vice versa*) or exchanging one biosimilar with another of the same reference product (if done by the prescribing physician, termed “switching,” or if done by the pharmacist, termed “substitution”) (1) should be addressed in a clear and unambiguous manner. Contrary to the evaluation and approval of biosimilars, which is generally centrally organized, decisions related to prescribing practices of approved medicines, including on interchangeability, fall under the responsibility of the individual EU Member States (1). As such, EMA has no official position or does not make recommendations on the interchangeability of biosimilars with their reference product (1, 34). The vacuum of guidance on EU level in this regard may be understood by some as a lack of crystallization of regulatory knowledge and endorsement of the safety of switching a reference product to its biosimilar or *vice versa*.

It is essential for healthcare professionals and patients to have access to trustworthy information about biosimilars, and their use. Regulators, as trusted and unbiased stakeholder, have a crucial role in providing this type of information. The availability of guidance and clear position statements on interchangeable use, including switching and substitution practices, from medicines agencies about biosimilars may be especially important to build confidence in biosimilars and enable their appropriate use.

The aim of this study is 2-fold. First, we aim to analyse how regulators on pan-European and national level provide information and guidance on biosimilars and their use, with a focus on guidance related to interchangeability, switching, and substitution. Second, we explore the perspective of two demand side stakeholder groups; healthcare and pharmaceutical industry professionals, on the role that regulators have in enabling acceptance and use of biosimilar medicines. In Box 1, an overview of the study highlights is shown.

Box 1 **Study highlights.**

**What is already known about the topic?**

- The EU pioneered the regulation of biosimilars, with establishing a framework for their evaluation and approval in 2004. Since then, the EU has approved over 65 biosimilars, the highest number of biosimilar approvals worldwide.
- While the evaluation and approval of biosimilars generally takes place on EU centralized level, guidance on their use (including guidance on interchangeability, and its related practices switching and substitution) is a responsibility of the individual Member States.
- Biosimilar use has been limited in some healthcare systems, which in part may be attributed to a variable understanding about the science and regulation underpinning their safe use among stakeholders. Moreover, healthcare professionals and patients have questions on the interchangeable use of biosimilars, and its related practices switching (exchange by the prescriber) and substitution (exchange at the pharmacy), and require guidance from regulators in this regard.
- The availability of information on biosimilars and clear regulatory position statements on interchangeability, including switching and substitution practices, is important to build confidence in biosimilars and inform healthcare professionals and patients on their appropriate use in clinical practice.

**What does the study add to existing knowledge?**

- This article reports results from a comparative review of the biosimilar information and position statements from the EMA and national regulatory agencies, complemented with qualitative insights from interviews with healthcare and pharmaceutical industry professionals on the role of European and national regulators.
- The results of this study reveal that information from national medicines agencies on biosimilars, and also guidance related to interchangeability, switching and substitution, differs considerably across Europe in terms of availability, extent, and content. Study results indicated that strong involvement in EU-level biosimilar regulatory activities (i.e., as national rapporteur/co-rapporteur for biosimilar MAA or member of the BMWP) seemingly correlates with the availability of more elaborate information and guidance on the national level.
- Important opportunity exists to expand biosimilar information on Member State level, as ~40% of national medicines agencies does not offer any biosimilar information or guidance on their use at present. Existing, EU developed healthcare professional and patient information materials can be leveraged more actively in this regard.
- Without the aim of interfering with local switch and substitution practices, regulators should collaborate to create a unified EU scientific position on the interchangeability of biosimilars, to unambiguously inform healthcare professionals, policy makers and patients with biosimilar use in clinical practice.

**What insights does the paper provide for informing health care-related decision making?**

- This study provides insight on the information and positions that European and national regulators provide on biosimilars and their use, and puts forth considerations on how regulatory action can further enable stakeholder trust in and use of biosimilar medicines.
- Findings may inform decision makers and healthcare professionals with the continued use and informed integration of biosimilars in healthcare systems and clinical practice.

*BMWP, Biosimilar Medicinal Products Working Party; EMA, European Medicines Agency; EU, European Union; MAA, Marketing Authorization Application*.

## Methods

A mixed methods design was employed, consisting of (i) a review and comparative analysis of available regulatory information on biosimilars in Europe and (ii) semi-structured stakeholder interviews to gain qualitative insights.

### Review and Comparative Analysis of Information and Position Statements From EMA and National Medicines Agencies About Biosimilars and Their Use

To analyse the availability, type, and extent of information and guidance provided by the European and national medicine agencies on biosimilars, the EMA and the national competent authority (NCA) websites in Europe were reviewed for content on biosimilars. Websites of the EMA and NCAs of the 27 EU Member States, the European Economic Area (EEA) (Norway, Liechtenstein and Iceland) and the UK, were screened (31 European countries in total). NCA websites were identified via the list provided on EMA's website and screened up to March 2019 (35). The overview of consulted NCA websites can be found in Supplementary Table S1. For countries for which two agencies were listed, information was integrated and counted as one in the results section. In total, 36 websites were screened for biosimilar information, both in English and with translated terms in the local language. Non-English retrieved information was translated to English with the help of an online text translator. Identified information was extracted based on a predefined set of parameters and subsequently tabulated in Microsoft Excel.

**Figure 1:**
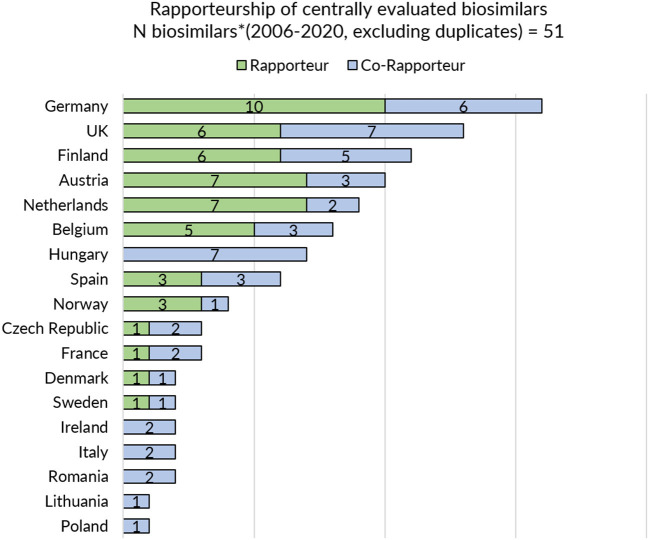
Rapporteurship of centrally evaluated biosimilars. *Biosimilars that received marketing authorization or received a position opinion and were pending EC decision between 2006 and 2020 were considered. Products that were withdrawn post-authorization were excluded. Duplicates were excluded.

Next, a sub analysis was conducted to explore a potential positive correlation between the information and guidance provided on biosimilars on a national level and the country's involvement in EU-level biosimilar regulatory activities. In order to assess the latter, countries' representation in the EMA's Biosimilar Medicinal Products Working Party (BMWP) and their involvement in the central evaluation of biosimilars was reviewed. To this end, the publicly available information on the composition of the BWMP was consulted (overview provided in Supplementary Table S2) and the European Public Assessment Report (EPAR) of every centrally approved biosimilar with a valid marketing authorization was screened for information on rapporteur and co-rapporteurships (overview provided in Figure 1). The analysis covered biosimilars that received marketing authorization or a positive opinion pending EC decision between 2006 and 2020. Products that were withdrawn post-authorization and duplicate marketing authorizations were excluded.

### Qualitative Stakeholder Interviews

To elicit qualitative insights, needs and proposals regarding regulatory guidance and information dissemination for biosimilars, exploratory semi-structured interviews (*n* = 14) were conducted with two European demand-side stakeholder groups, i.e., healthcare professionals and pharmaceutical industry representatives. A purposive sample of interview participants was gathered via professional organizations and via the network of the research group. A topic guide was designed, evaluated and piloted with one participant per stakeholder group. Supplementary Table S3 in Supplementary Information provides an overview of the topics discussed during the interviews. Interviews were conducted face-to-face or via teleconference between February 2019 and April 2019. Interviews were audio-recorded and transcribed *ad verbatim* with the written informed consent of the participant. Interviews were conducted until data saturation (36). The *ad verbatim* transcripts were pseudonymized, coded and thematically analyzed according to the thematic framework approach, using NVivo qualitative data analysis software (37).

## Results

### Comparative Analysis of Information and Position Statements From EMA and National Medicines Agencies About Biosimilars and Their Use

#### Biosimilar Information and Education Resources for HCP and Patients From Regulators Across Europe

**Figure 2:**
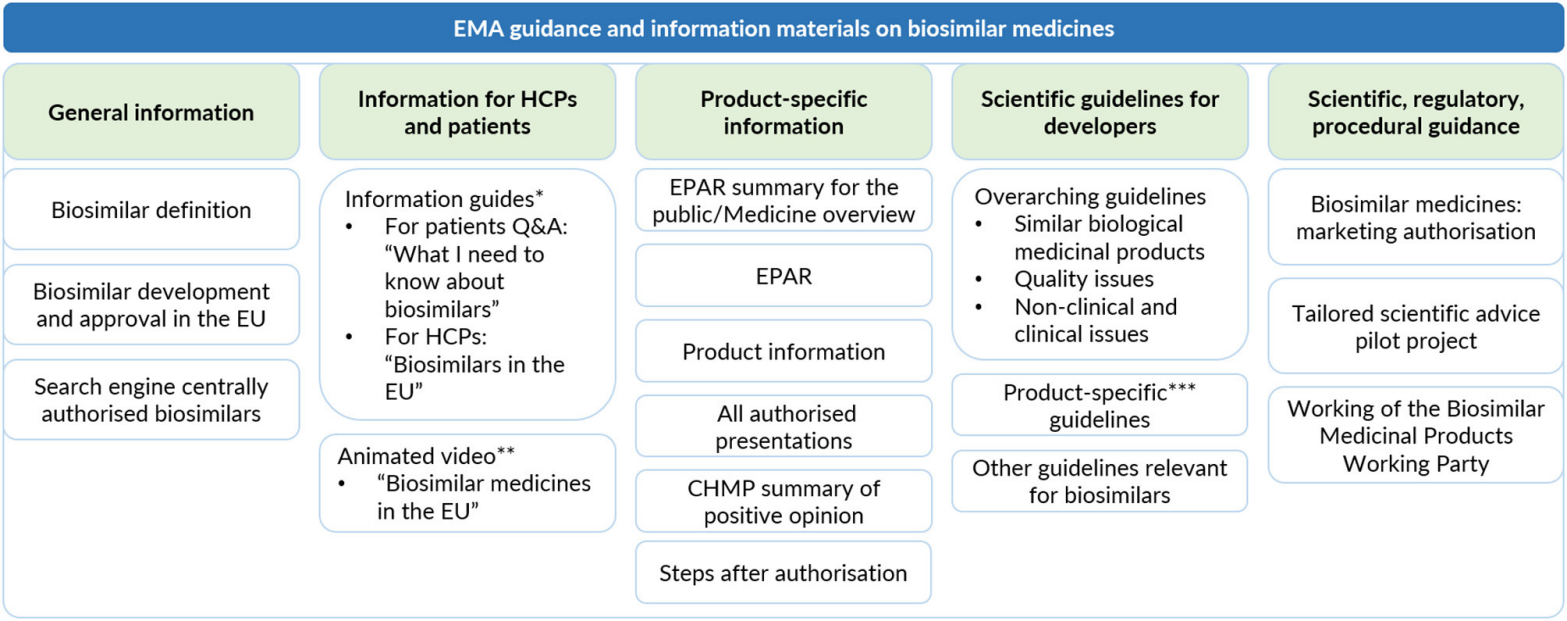
Overview of EMA's information material and guidance documents on biosimilar medicines. *Available in 23 official EU languages. **Available in English and other EU languages (Dutch, English, French, German, Italian, Polish, Portuguese, Spanish). ***For recombinant granulocyte-colony stimulating factor, low-molecular-weight heparins, recombinant human insulin and insulin analogs, interferon beta, monoclonal antibodies, recombinant erythropoietins, recombinant follicle-stimulating hormone, somatropin.

Besides providing scientific, regulatory, and procedural guidance as part of one of the Agency's principal responsibilities as regulatory authority, the EMA developed together with the EC educational materials on biosimilars for healthcare professionals and patients (1, 27, 38). Both the information guide for healthcare professionals and the information leaflet for patients were made available in all 23 official EU languages. In addition, an animated educational video “*Biosimilar medicines in the EU*” was developed and translated into multiple EU languages. The EMA's website has a dedicated landing page for biosimilar related information, which includes hyperlinks to these educational materials and other relevant information resources, on biosimilars in general and on a product-specific level (38). Figure 2 provides an overview of the information and guidance that is provided by EMA on biosimilar medicines.

**Figure 3:**
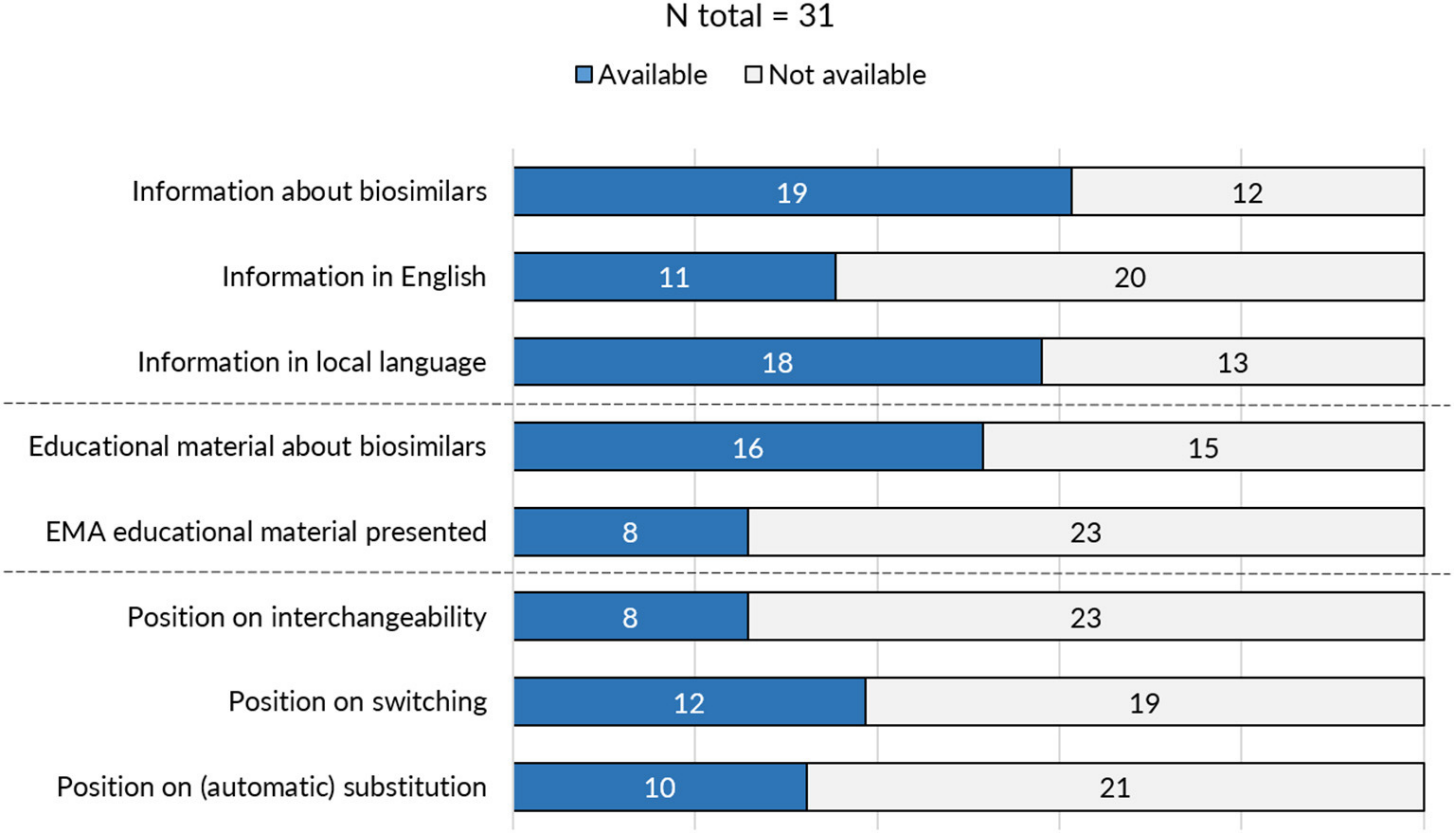
Overview of biosimilar information and guidance provided by national medicines agencies across Europe.

On the level of the individual Member States, the provision of information and educational materials on biosimilars varied between countries. Surprisingly, of the 31 medicines agencies, only 19 offered information about biosimilar medicines (Figure 3). Of the national medicines agencies that offered information about biosimilars, all except for Austria, Malta, and Norway, also provided educational resources on biosimilars. The type of educational material displayed differed across agencies. Either these were designed by the NCA itself or originated from the EMA/EC prepared stakeholder information material. Eight agencies relied fully or in part on one or multiple of the EMA's/EC's educational resources on biosimilars. Table 1 presents an overview of the availability of information and educational materials on biosimilars by national medicines agencies. Educational materials provided by regulators included videos, radio spots, booklets, workshops, conferences, position papers, campaigns, and presentations. An overview of educational materials and initiatives per NCA is presented in Supplementary Table S4.

**Table 1:** Overview of availability of biosimilar information and guidance provided by national medicines agencies.

**Country**	**Information on biosimilars**	**Educational material**		**Interchangeability position**	**Switching position**	**Substitution position**
		**Available**	EMA/EC material^*^			
Austria	Y	N	N	N	N	N
Belgium	Y	Y	N	N	Y	Y
Bulgaria	N	N	N	N	N	N
Croatia	Y	Y	Y	Y	Y	Y
Cyprus	N	N	N	N	N	N
Czech Republic	N	N	N	N	N	N
Denmark	Y	Y	N	N	Y	N
Estonia	N	N	N	N	N	N
Finland	Y	Y	N	Y	Y	Y
France	Y	Y	N	Y	N	N
Germany	Y	Y	N	N	Y	Y
Greece	N	N	N	N	N	N
Hungary	Y	Y	Y	N	N	N
Iceland	Y	Y	Y	N	N	N
Ireland	Y	Y	N	Y	Y	Y
Italy	Y	Y	Y	Y	Y	N
Latvia	N	N	N	N	N	N
Liechtenstein	N	N	N	N	N	N
Lithuania	N	N	N	N	N	N
Luxembourg	N	N	N	N	N	N
Malta	N	N	N	N	N	N
Netherlands	Y	Y	Y	Y	Y	Y
Norway	Y	N	N	N	Y	Y
Poland	N	N	N	N	N	N
Portugal	Y	Y	Y	N	Y	Y
Romania	N	N	N	N	N	N
Slovakia	Y	Y	Y	N	N	N
Slovenia	N	N	N	N	N	N
Spain	Y	Y	Y	N	N	N
Sweden	Y	Y	N	Y	Y	Y
UK	Y	Y	N	Y	Y	Y

* *EMA/EC's HCP and/or patient guide and/or animated video presented on website. Y; available, N; not available*.

#### Regulatory Position Statements on Interchangeability, Switching, and Substitution

As prescribing practices and advice to prescribers falls within the remit of the individual Member States, there is no official position or recommendation on the interchangeability of biosimilars at the EU level (1). However, a group of regulators, members of the Biosimilar Medicinal Products Working Party (BMWP), EMA/CHMP's European expert group on biosimilars, published under personal name an article stating that biosimilar products authorized in the EU are interchangeable (33). More in particular, they conclude that the demonstration of biosimilarity, together with post-marketing surveillance, adequately ensures interchangeability of EU-approved biosimilars under supervision of the prescriber. Further, they mention that, if needed, the patient should receive proper training on the administration of the new product (33).

In the EMA/EC biosimilar information guide for healthcare professionals, clear definitions have been provided on interchangeability, switching and substitution. The guide goes further with stating that “*there is no reason to believe that harmful immunogenicity should be expected after switching between highly similar biological medicines*.” Furthermore, it includes that “*any decision on switching should involve the prescriber in consultation with the patient, and take into account any policies that the country might have regarding the prescribing and use of biological medicines*” (1). In the EC's patient Q&A leaflet on biosimilars, mention is made that “*switching is a growing practice in some Member States*” (27).

In 2019, the International Coalition of Medicines Regulatory Authorities (IMCRA), bringing together heads of 29 medicines regulatory authorities from different regions of the world—of which the EMA and EU national medicines agencies are member—released a position statement for healthcare professionals aiming to provide them with assurance and confidence in biosimilar use. On switching, they comment that it is “*an accepted clinical practice in many countries*” (3). Table 2 provides an overview on available statements and guidance by regulators and regulatory agencies at the European level.

**Table 2:** Positions about biosimilar interchangeability, switching, substitution by regulators at the European level.

**EMA/EC HCP and patient biosimilar information guides (1, 27)**
- The HCP guide explains that EMA does not regulate interchangeability, switching, or substitution as these practices are under the responsibility of Member States. As such, no formal position is provided about interchangeability or substitution. - However, some supportive messages dispelling concerns about switching were included: • HCP guide: “There is no reason to believe that harmful immunogenicity should be expected after switching between highly similar biological medicines,” “if a patient is switched from one biological medicine to another with the same active substance, it is important to record the tradename and batch number for each of the medicines,” “any decision on switching should involve the prescriber in consultation with the patient, and take into account any policies that the country might have regarding the prescribing and use of biological medicines.” • Patient Q&A: “It is possible to switch from a biological reference medicine to a biosimilar medicine and this is a growing practice in some Member States. Any decision on switching should be taken by your doctor in consultation with you, and taking into account any policies that your country might have regarding the use of biological medicines.”
**Scientific publication by group of individual European regulators Kurki et al. (33)—“Interchangeability of Biosimilars: A European Perspective”**
- “Because of the high similarity, there is no reason to believe that the body's immune system would react differently to the biosimilar compared with the original biological upon a switch. This view is supported by the current experience with biosimilars on the market and by literature data. In our opinion, switching patients from the original to a biosimilar medicine or vice versa can be considered safe.” - “Our conclusion is that biosimilars licensed in the EU are interchangeable.”
**International Coalition of Medicines Regulatory Authorities/ICMRA (3), which includes EMA, EC DG SANTE and several national medicines agencies as members—statement about confidence in biosimilar products (for healthcare professionals)**
- “Changing between originator and biosimilar (i.e., a prescribing healthcare professional transferring a patient on treatment from one medicine to another) is an accepted clinical practice in many countries.” - “Some countries have regulatory frameworks that permit substitution at the pharmacy level (i.e., without intervention by the prescriber) under certain conditions.”
**Scientific publication by group of individual European regulators Kurki et al. (64)—“Safety, Immunogenicity and Interchangeability of Biosimilar Monoclonal Antibodies and Fusion Proteins: A Regulatory Perspective”** - “Our study, together with previous reports, suggest that concerns regarding immunogenicity upon switches are unfounded. Thus, systematic switch studies are not needed.” - “Interchangeability of EU-licensed biosimilars has been demonstrated. Thus, automatic substitution at the pharmacy level is, in principle, possible. From the European perspective, substitution should be tailored to the local circumstances, such as methods for traceability, the need for training of patients and pharmacy personnel, and the switch protocol, including the timing of/interval between switches and price differences triggering a substitution.”

**Figure 4:**
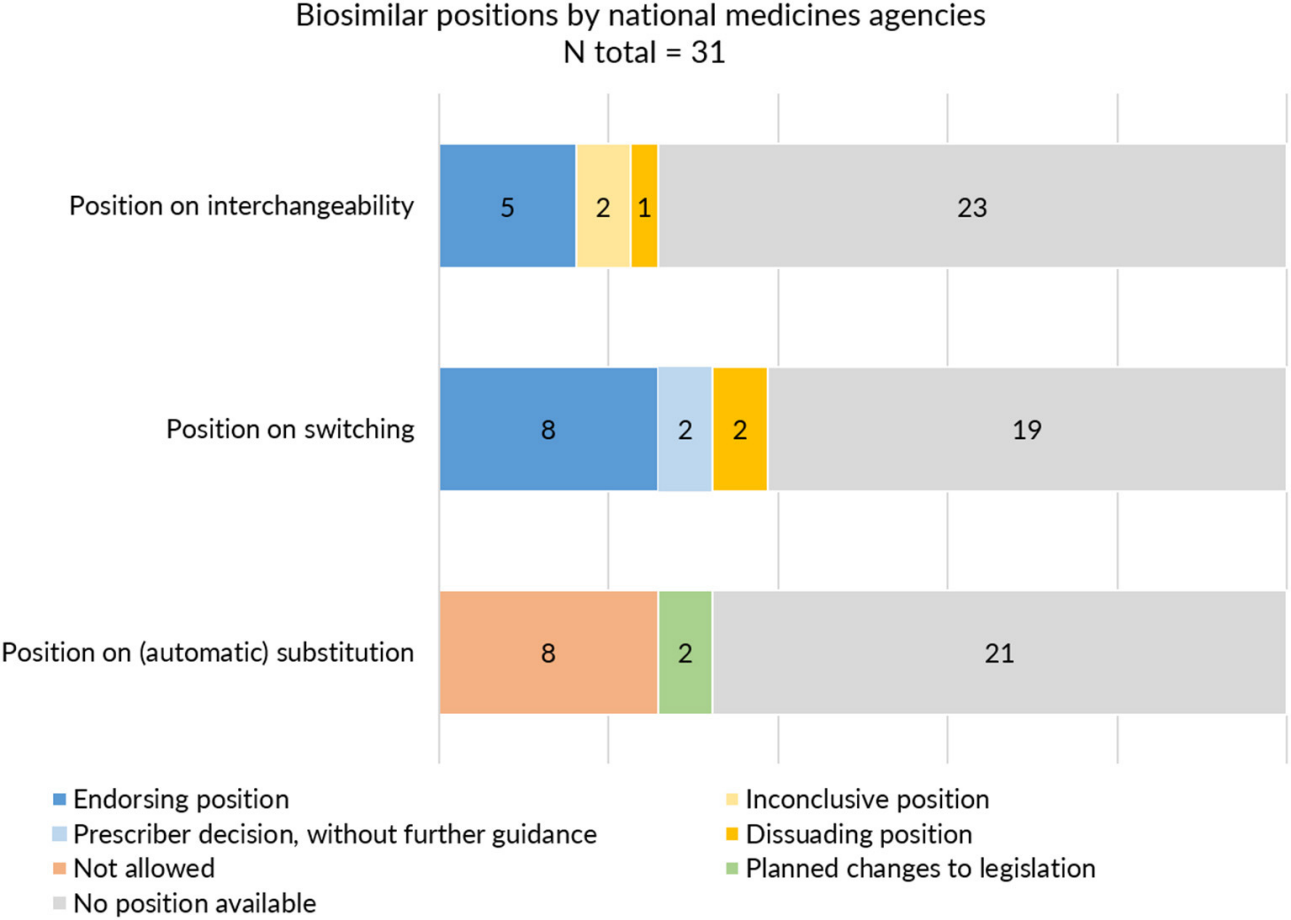
Interchangeability, switching and substitution: type of positions provided by European medicines agencies.

On the level of the individual Member States, positions on interchangeability, switching, and substitution for biological medicines were not provided by all and varied in extent and content. Despite this being the responsibility of the Member States, guidance about interchangeability, switching, and substitution was absent from more than half to two third (60–74%) of national medicines agencies (Table 1). Figure 4 provides a schematic overview of the type of positions provided by national medicines agencies on interchangeability, switching, and substitution.

With regards to interchangeability, only eight out of 31 medicines agencies offered an explicit statement. When available, positions varied between agencies in terms of message. While some regulatory agencies endorsed interchangeability of biosimilars, such as the Finnish Medicines Agency (FIMEA) or the Dutch Medicines Evaluation Board (MEB) providing already an explicit position in 2015, others provided a more reserved statement (Supplementary Table S5). The Swedish agency was more cautious, stating that “*the risk of immunological reactions during frequent changes is incompletely elucidated*.” Contrary to most agencies which generally provided a brief statement of a few sentences, FIMEA published a dedicated four page report to define their position on the interchangeability of biosimilars, providing information on context and explaining the scientific rationale behind their position (39).

With regards to switching, 12 NCA websites provided an explicit position. In general, switching statements were comparable between NCA websites, commenting that relevant changes in treatment outcomes are not expected upon switching from the reference product to a biosimilar or *vice versa*. Despite being generally supportive, different nuances were made. Some agencies focussed mainly on reassuring the safety of switching by for example referring to the growing availability of clinical switch data. Others underlined the authority of the prescribing physician in making switch decisions without providing further guidance. Two agencies explicitly discouraged back and forth switching between biosimilars and their reference product. Only three national medicines agencies specifically made reference to biosimilar to biosimilar switching (Supplementary Table S6).

In the context of (automatic) substitution, only 10 national medicines agencies provided a clear position of which most indicating automatic substitution to be not allowed. A few countries pointed toward foreseen changes in legislation to eventually permit automatic substitution of biologicals (of certain product types or under certain conditions) (Supplementary Table S7). In Germany, substitution of biosimilars was already possible, but limited to the substitution of “bioidenticals” or “duplicates,” i.e., biosimilars made by the same manufacturer, which have been licensed under a different brand name. More recently, a new legal framework has been introduced in the context of the “*Gesetz für mehr Sicherheit in der Arzneimittelversorgung* (GSAV)” or “*law for more safety in the supply of pharmaceuticals*,” broadening the application of automatic substitution of biologicals beyond bioidenticals (40). The German Statutory Health Insurance (G-BA) is responsible of translating this into practice, with offering two sets of guidance: one toward physicians with details on how to switch and one toward pharmacists, providing a positive list of biosimilars eligible for automatic substitution. The change is planned to come into effect in 2022 (40). Also in Norway, the possibility for automatic substitution of biologicals is being considered, with the national medicines agency proposing the Pharmacy Act § 6-6, which forms the basis for generic pharmacy substitution, to be changed to allow automatic substitution for biologicals (41). Table 3 provides an overview of automatic substitution practices for biological medicines across Europe.

**Table 3:** (Automatic) substitution for biological medicines in Europe: an overview of practices.

**Allowed (under specific conditions)**	**(Planned) changes to legislation**	**Not allowed**		**No info**
France^a^ Hungary^a^ Latvia Lithuania Poland^b^	Germany^d^ Norway^e^	Austria Belgium Croatia Czech Republic Denmark Finland Greece Iceland Ireland	Italy Malta Netherlands^c^ Portugal Romania Spain Sweden UK	Bulgaria Cyprus Estonia Liechtenstein Luxembourg Slovakia Slovenia

a Authorized by law under specific conditions (e.g., only for treatment naïve patients), but not implemented in practice*.

b *Automatic substitution is not recommended, but due to a lack of regulation or specific guidance, automatic substitution may occur*.

c *For insulin biosimilars, insurance companies are increasingly forcing pharmacies to substitute to the biosimilar*.

d *New legislation planned [GSAV: Gesetz für mehr Sicherheit in der Arzneimittelversor-gung], that will allow biologicals to be substituted at pharmacy level*.

e *Proposal to alter Pharmacy Act § 6-6 (basis for generic (automatic) substitution in pharmacies), eventually permitting automatic substitution of new classes of medicinal products, e.g., biological drugs*.

*Sources: consulted NCA websites and (40–42)*.

In general, regulatory medicines agencies from Western and Northern European countries appear to provide more elaborate biosimilar guidance. Strong representation of Member States in EU level regulatory activities for biosimilars such as involvement in EMA's BMWP [with members from Austria, Belgium, Denmark, Finland, France, Germany, Ireland, Sweden, and the Netherlands (43), overview in Supplementary Table S2] and rapporteur or co-rapporteurship (Figure 1: Germany, UK, Finland, Austria, and the Netherlands have been most frequently in the lead) in biosimilar evaluation appears to have translated in more elaborate and outspoken regulatory biosimilar guidance on a national level.

### Qualitative Insights From Semi-structured Expert Interviews

Fourteen expert stakeholders participated in a semi-structured interview. An overview of participant characteristics is shown in Supplementary Tables S8, S9 in Supplementary Material. The interview results are structured according to the five main themes derived from thematic analysis of the interview transcripts.

#### EMA Leading the Way in Guidance and Communication About Biosimilars

EMA's efforts toward improving stakeholder understanding about biosimilars were recognized, with several interviewees underlining the positive evolution in terms of stakeholder outreach. Especially, the EMA/EC information guides for healthcare professionals and patients were perceived as reference documents in the field, which helped to inject trust in biosimilars and disseminate clear messages toward the medical community.

“*In the past years, the way EMA is communicating and putting documents on their website, you see that they try to be as clear and explicit as possible also in some kind of lay language. They try to convert their regulatory text toward the audience of prescribers and patients. That is positive in my opinion.” (HCP7)*

“*They [EMA] have been successfully convincing the physicians' community in general that the way the evaluations have been done is sufficiently efficacious. That was at the beginning the problem, because we were not familiar with the kind of investigation that the EMA proposed.” (HCP4)*

At large, EMA was considered to lead the way in terms of biosimilar regulatory science and communication: “*Once they make a decision or statement the rest will follow*. *So it is important that organizations such as EMA play their role in informing the general public*.” *(I2)* Strong regulatory communication was considered especially important in the context of dispelling misinformation about the underlying science of biosimilars.

Also, the publication of scientific articles about biosimilars by European regulators was recognized to have been helpful to update the medical community. However, some interviewees mentioned that it was not always clear to them if these presented the position of the individual authors or that of the agency. EMA's website was considered to be a rich source of information on biosimilars. Yet, despite the fact that the website has a dedicated page on biosimilars, several interviewees cautioned that relevant information may not be easy to retrieve for healthcare professionals and patients. In addition, several interviewees argued that the role of EMA may not be well-known by all, recommending to increase awareness about the EMA and its activities in general.

Some interviewees mentioned that promoting biosimilars may go beyond the remit of the EMA, and considered it not to be the EMA's responsibility to take up an active role in stakeholder education. Others argued that consolidating efforts at central level in terms of developing stakeholder guidance may positively contribute to homogenous messaging across Member States. A few interviewees remarked that while information should be made available at EU level, its dissemination is the responsibility of the NCA's and professional organizations, who should subsequently make use of the information to inform stakeholders more locally.

“*EMA is the reference, and they have a role to be transparent, but I do not think it is up to them to insure dissemination of this information. …They are doing more and more, but it is not their job to make sure that all HCPs and patients know and understand exactly what a biosimilar is. I think there is a lot to do at national level and in the professional organizations as well.” (I7)*

#### The European Public Assessment Report as Transparent Tool on Biosimilar Evaluation—Is It Fit for Purpose?

Interviewees deemed the EPAR an important tool to transparently inform about the regulatory evaluation and decision-making to approve or refuse a market authorization for a given medicine. The EPAR was considered to be especially useful by pharmaceutical industry interviewees as an instrument for them to learn about competing products. Although interviewees agreed that the EPAR is important to provide insight in product evaluation, some remarks were made. First, interviewees noted that the level of transparency provided by the EPAR may depend on the time of publication of the EPAR, with newer EPARs being more detailed and structured than older ones. Second, enhancing the level of substantiation provided in the EPAR was noted as a point for improvement. Reading the EPAR was considered requiring the ability to “read between the lines,” and interviewees would like to see more justifications regarding the outlined decisions (e.g., providing more in depth reasoning why something was considered acceptable or not).

While the EPAR was generally considered fit for purpose for expert and industry stakeholders, the document was considered too complex and long to serve as informational or educational instrument to inform healthcare professionals with their daily practice. Most interviewees believed that individual physicians are not likely to use the document: “*Transparency is there, but it is not because you have a PDF online that people will read it. If you're a prescribing physician, you won't read the EPAR I think.”* (I4) It was also mentioned that healthcare professionals are generally not aware about the existence of the EPAR. Several interviews argued that a shortened version, in addition to the full EPAR, should be made available. It was suggested that such summary should not only provide general information on the product (as is currently made available in the Medicine Overview document), but include a conclusion on why the biosimilarity assessment was concluded to be positive, equipping stakeholders with the rationale behind EMA's evaluation and opinion. An interviewee pointed toward the structural change in EPARs of more recently approved biosimilars, which include a specific concluding section on biosimilarity: “*If you just want to grab the main points about biosimilarity, it is easy because you can go directly to the biosimilarity section*” *(HCP2)*.

#### European vs. National Responsibilities

The provision of clear information and consensus papers at EU level was mentioned to be important to help steer and shape initiatives at national level. Clear EU-information and guidance may spur national agencies to action, and closer cooperation between EMA and the NCAs was advocated in this regard. Filling the gap between the EMA and national medicines agencies and strengthening the guidance by the latter was considered important. NCA guidance was believed to have a more direct and tangible impact on activities at the national level, and NCA's may coordinate more easily with local stakeholders.

“*I think that national competent authorities play a more important role because they have more visibility in their respective countries” (I1)*

Interviewees argued that NCAs should explore ways to provide more dynamic information opposed to short, static information on the NCA's website: “*It should be more dynamic as opposed to the way it is put now on their website.” (HCP7)*. Suggestions included the establishment of a Q&A platform, and videos where patients, physicians, and heads of the medicines agency etc. could speak up on the use of biosimilars. Several interviewees pointed to the fact that information provided by NCAs appears to be difficult to retrieve in some cases, which may be especially hindering for non-experts in the field.

#### National Competent Authorities to Address Interchangeability, Switching, and Substitution

Interviewees pointed toward the sometimes limited and variable guidance between NCAs regarding the use of biosimilars:

“*NCAs have in general not been clear on how biosimilars could be integrated into the treatment of patients. No one had clearly communicated that it [interchangeable use] is a possibility. It is a maze for a non-expert to understand what they should do in their country. You have to go, like trying to find the Da Vinci code, through details, websites and try to figure out what the recommendations are.” (I4)*

“*Some agencies in Europe were more pro-active in this regard. I think it is also linked to having a strong advocate in the country.” (I4)*

In addition, some interviewees found positions to be too implicit. In this context, it was mentioned that positions appear to largely address only a single switch from reference product to biosimilar:

“*It is important to provide information more extensively and more precisely in the future. Especially more guidance is important for situations like multiple switching” (HCP5)*

Another interviewee mentioned “*NCAs could be more proactive on that, but we have many sources of information that we use to make our own decisions” (HCP4)*.

Some interviewees argued for a more central coordination on biosimilar-related information and position statements, to ensure convergence. Some mentioned that EMA should publish guidance about interchangeability as the limited and heterogeneous guidance on Member State level may lead to confusion. Others anticipated it difficult to develop guidance that would be accepted across Europe.

#### Informing and Educating Stakeholders About Biosimilars—A Collaborative Effort Between Regulators and Scientific Stakeholder Societies

The collaboration between EMA and healthcare professional stakeholder organizations in the context of biosimilar information development was recognized as positive. Interviewees stressed the importance of joining forces, explaining that healthcare professional stakeholder organizations can help translate and tailor regulatory information to the needs of their members. Healthcare professional associations were considered to be crucial in conveying trust and should be considered as an active link between EMA and the healthcare professionals. A few interviewees mentioned that having information on EMA's website is especially important for scientific associations for them to disseminate it, rather than for the individual physician to consult EMA's website directly. Well-informed physicians may then in turn inform their patients.

“*It is crucial that these kinds of scientific associations endorse the regulatory approval and try to express that endorsement toward their members*.” *(HCP7)*

## Discussion

Access to trustworthy and transparent information about biosimilars and clear guidance on their use is essential to improve understanding on biosimilars and appropriately inform healthcare professionals and patients regarding their implementation in clinical practice. This study aimed to assess how regulators, both on a central and national level in Europe, provide information and guidance about the evaluation and use of biosimilars, with a specific focus on guidance related to interchangeability, switching and substitution, and how this is perceived by external demand-side stakeholders. To this end, both a review and comparative analysis of publicly available information and position statements regarding biosimilar use by EMA and national medicines agencies and semi-structured expert interviews with healthcare and pharmaceutical industry professionals were conducted.

### Regulatory Information and Positions on Biosimilars and Their Use: Untapped Opportunities at the National Level and a Need for Harmonization

While biosimilar evaluation and approval relies on a solid centrally coordinated European regulatory pathway, with external stakeholder dissemination strategies to explain the underlying science underpinning their evaluation and use (19, 26, 33, 44, 45), this study found that at the national level the information and guidance available on biosimilars considerably varies between medicines agencies. Information on biosimilars, and positions on their use, i.e., on interchangeability, and the associated practices of switching and substitution, are not consistently available and vary in extent and content.

This gap in consistent information on biosimilars at the national level may be explained by the fact that providing guidance on interchangeability, switching, and substitution falls outside the otherwise centrally organized evaluation and approval of biosimilars, and is managed at Member State level. These decentralized responsibilities appear to have been addressed to different degrees across Member States. Overall, regulatory information provision on biosimilars appears to have operated at different speeds between the EU and the national level. While prescriber practices across Member States are expected to show a certain degree of heterogeneity as these practices are shaped in the context of their respective healthcare systems and medical culture (i.e., frameworks to allow for physician-led switching and/or pharmacy-led substitution), a uniform position from a scientific viewpoint on biosimilar interchangeability is to be expected. The observed heterogeneity between positions of national regulatory agencies, together with the absence of a clear EU position on interchangeability, may suggest a lack of regulatory and scientific clarity on the safety of an exchange between reference product and biosimilar. This poses a source of confusion among stakeholders and is argued to have been amplified by the (originator) pharmaceutical industry (9, 21).

Besides clear regulatory guidance on biosimilar use, clear regulatory information regarding biosimilars, and the science underpinning their evaluation and safe use is believed to be essential to build stakeholder confidence. Whilst the precise impact of regulatory information and guidance on biosimilar acceptance is hard to isolate from other drivers at play, its availability is essential to provide stakeholders with accurate and trustworthy facts, and dispel misinformation in the debate. The concept of biosimilarity, and especially the fit-for-purpose reduction of clinical studies is difficult to explain to clinicians who are accustomed to rely on clinical trials in the context of new drug development. The mantra “*similar but not the same*” and “*subtle differences*” that trigger immune reactions has evoked considerable uncertainty and propelled investments in extensive switch studies (46).

Furthermore, regulatory information forms the basis for subsequent coherent and accurate information dissemination on biosimilars and their use more locally. It is exceptional that regulators have to defend the quality, safety, and efficacy of medicinal products licensed by EMA. However, it is necessary in the context of biosimilars in order to establish trust on and dispel uncertainties regarding the robust EU regulatory framework underpinning their safe use.

### The Interchangeable Use of Biosimilars

The discussion on whether or not a biosimilar can be safely interchanged with the reference product or other biosimilars has persisted since their introduction (47, 48). This discussion touches upon how biosimilars can be used in clinical practice, especially so for biosimilars that are intended for used in a chronic treatment setting, and is as such essential to address. While concerns were raised that an interchange between non-identical biologicals might result in an increase in immunogenicity, this has not been observed in clinical practice and the theoretical basis that this would occur has been considered to be weak (33, 49). Based on the available clinical data from over a vast body of clinical switch studies, no apparent signals were detected to assume that switching would be associated with any major efficacy, safety, or immunogenicity concerns (50, 51). For biosimilars that met EU regulatory requirements, it is considered unlikely that the body's immune system would react differently to the biosimilar upon a switch since comparable structure and immunogenicity has been demonstrated between the biosimilar and its reference product (33, 49). Clinical data continue to emerge, also on multiple switching, and switching has been routinely adopted in clinical practice in several healthcare settings across Europe (27). While the scientific discussion on switching from reference product to biosimilar has been largely settled, questions on multiple switching and switching between biosimilars of the same reference product emerged, and healthcare professionals advocate for more scientific and regulatory clarity in this regard to support them with the appropriate use of biosimilars in clinical practice (52).

Whereas, in Europe switching generally takes place under supervision of the prescriber and legislation stems from a period before biosimilar market entry, some countries are planning to allow for substitution of biologicals at the pharmacy level (40, 53). The translation of substitution of biologicals in practice would involve an assessment of substitutability on product-specific level by the national medicines agency, upon which the biosimilar could be included in an “exchange” or “substitution list” (40, 53). In this context, it will be essential that community pharmacists are well-prepared and trained to appropriately counsel patients with such a transition. The pharmacist must be familiar and confident in biosimilar use to mitigate for possible nocebo effects, and trained to counsel the patient with a possibly new injection device that such an exchange may entail (20, 47, 54). While this will require efforts, trained pharmacists may be a reliable source of clear information on biosimilars and their use.

It is important to note that regulatory approaches for biosimilar interchangeability vary across the globe, which may also be a contributing factor to misunderstanding and uncertainty among policy makers and the clinical community. Whereas, interchangeability assessment is not part of regulatory biosimilar evaluation in Europe or in Australia, in the US the US Food & Drug Administration (FDA) has a dedicated regulatory pathway for biosimilar interchangeability designation (34, 47, 55, 56). This interchangeability designation regulates automatic substitution, i.e., biosimilars that receive interchangeability designation may subsequently be substituted by the pharmacist without intervention of the prescriber, if also in line with state law (see Supplementary Box S1 in Supplementary Information) (57). Given these differences in regulatory approaches, it is important to consistently position the discussion in its correct geographical context to mitigate for possible misconceptions (21).

### A Call for Strengthened Biosimilar Guidance on the National Level and a Unified EU Scientific Position on Interchangeability

The EMA has expressed its continued commitment in developing actions to reinforce trust and confidence in biosimilars (58). In EMA's *Regulatory Science 2025 Strategic reflection*, promoting the availability of biosimilars and supporting their uptake in healthcare systems was included as a core recommendation to advance patient-centered access to medicines. This point was again reiterated in the EMA and HMA Network Strategy to 2025 (58, 59).

Europe has been leading the way in the field of biosimilars since the introduction of the first regulatory pathway for biosimilars in 2005, and the strong scientific and stakeholder outreach track record in this regard should be continued at the national level.

Three main recommendations are advanced:

(i) The availability of consistent one-voice information about biosimilars should be strengthened across national medicines agencies. For the latter, national regulators can leverage existing, EU developed healthcare professional and patient information materials locally. These materials have been made available in all 23 EU languages for the purpose of supporting consistent messages and education on biosimilars throughout the EU, and can be easily made available on national websites. In addition, several national agencies developed detailed stakeholder information about biosimilars, which may serve as a basis for other national medicines agencies (60, 61).

The scientific and regulatory knowledge and expertise with biosimilars that is consolidated at EMA and BMWP level could be leveraged to further aid initiatives at the national level. A closer collaborative framework between the EMA (BMWP, EMA Biosimilar Matrix) and the national medicines agencies could strengthen information dissemination from the central to the national level, and leverage and transfer EU level biosimilar expertise across the broader European regulatory network. Furthermore, closer collaboration between regulators may stimulate the exchange of biosimilar best practices among Member States, and result in coordinated action to respond to biosimilar misinformation and queries that emerge at the national level. In terms of concrete initiatives to foster this collaboration, the recently established Heads of Medicine (HMA) Biosimilar group, which is composed of representatives nominated by interested national medicines agencies and an EMA representative, is an important step and platform in this regard (62, 63).

(ii) Besides strengthening the availability of information and education on biosimilars at the national level, regulators should join forces and act swiftly to provide a unified and unambiguous scientific EU position on biosimilar interchangeability. The lack of EU-level guidance in this regard and the variation in positions from national medicines agencies across Member States might unintentionally suggest a lack of regulatory and scientific clarity on this. Guidance should include information on reference to biosimilar, biosimilar to reference and biosimilar to biosimilar switching.

In 2018, individual members of the BMWP paved the way for a scientific position beyond national Member State boundaries by conveying the European perspective with regards to interchangeability and the safety of switching in the form of a scientific publication published under personal name (33). A next step is now needed to clearly address the discussion on biosimilar interchangeability and switching from a formal regulatory point of view, and unambiguously inform healthcare professionals who are confronted with questions related to this in clinical practice. While a clear regulatory position is needed to provide guidance on the population level, it is up to the prescriber to decide on the suitability of an exchange on the level of the individual patient. Furthermore, it should be made clear that such a unified scientific position would not have the goal of intervening with the Member States' sovereignty regarding prescribing and dispensing practices. Policy decision regarding prescribing practices including switching and substitution should be made in the context of the local healthcare system, and such a unified position may inform healthcare decision makers in the development of policy measures related to biosimilar use. In a recent publication, a group of European regulators underwrote the importance of creating a common European position on biosimilar interchangeability with the aim of promoting rational use of biologicals (64).

Such unified position requires central coordination and cooperation between national regulatory agencies (47, 52). Also here, the recently established HMA Biosimilar group may play a vital role (63). NCAs could ask CHMP for a scientific opinion (referral) or HMA to issue a common scientific opinion. In addition to this, in the context of the European Commission's Pharmaceutical Strategy for Europe, it was announced that the topic of interchangeability will be addressed in the upcoming 2022 review of the European pharmaceutical legislation (65). These initiatives together may provide a timely and much needed momentum to unambiguously address biosimilar interchangeability on a European level.

(iii) To make reliable information on biosimilars more easily retrievable for stakeholders, a centralized, European-led online repository for healthcare professionals and patients on biosimilar medicines could serve as central go-to information hub, with one-voice, factual information on biosimilars that is in line with the latest scientific and regulatory experience. On a product-specific level, the EPAR may be leveraged more actively—and especially the dedicated discussion on biosimilarity which was part of a revision to increase more transparency on the assessment—by creating awareness on its existence (66–68).

### Informing Stakeholders Requires a Coordinated Multi-Stakeholder Effort

While regulators have an important role in providing clear information on biosimilars and the regulation and science underpinning their use, conveying trust in the use of biosimilars and effectively educating physicians and patients about biosimilars requires a multi-stakeholder effort. Besides regulatory authorities, professional stakeholder associations such as healthcare professional and patient organizations have an important role in informing and translating regulatory guidance to physicians, pharmacists, nurses and patients (9, 30).

The availability of clear regulatory information and guidance about biosimilars may form the basis of correct and unbiased stakeholder information, but—as also emphasized during the interviews—needs further active leveraging from stakeholder organizations to actually reach the healthcare professional and patient. It may be unrealistic to expect that busy clinicians regularly consult regulatory websites. Instead, they often rely on peer key opinion leaders in the field. As such, regulators should continue to seek collaboration with healthcare professional and patient organizations to effectively disseminate unbiased and correct information about biosimilars, on the European as well as on the national level (9, 30, 69).

### Strengths and Limitations of the Study

Based on a structured mapping of the available information from European regulatory agencies and qualitative stakeholder interviews, this study offers new and important insights on the European landscape of regulatory information and guidance on biosimilars and their use. However, some limitations need to be considered. The fact that some NCA websites offered information only or in part in the Member State's local language made the retrieval and extraction of relevant information complex. Websites were thoroughly scanned for biosimilar information with both English and local language translated terminology, but certain omissions cannot be excluded. Non-English retrieved information was translated to English with the help of an online text translator. This may have led to small differences in nuances of wording between original and translated position statements. Furthermore, the web-based screening allows to only collect and review information that is made publicly available on the websites of the regulatory agencies. Since the scope of this study was to investigate the guidance provided by regulatory authorities across Europe, it did not screen or evaluate guidance that local pricing and reimbursement authorities or ministries may have issued on the use of biosimilars.

The qualitative component of the research allowed to gather stakeholder insights and proposals on regulatory information and guidance dissemination for biosimilars and the role European and national regulators have in this regard. Interview participants were purposefully selected based on their expertise and pan-European and/or nation al insights on the study topic. It should be noted that—as with qualitative research in general—the findings are bound to the participant sample. While the qualitative part of the study focussed on the perspective of healthcare and industry professionals, future research could explore the perspective and needs of other stakeholders such as policy makers and patients. In addition, a study with European regulators may further distill actionable avenues forward from the perspective of the regulator.

## Conclusion

This study showed that regulatory information and guidance on biosimilars and their use, i.e., on interchangeability, and associated practices of switching and substitution, considerably varies across national medicines agencies in terms of availability, extent, and content. Untapped opportunity exists at the national level to expand and harmonize regulatory information and guidance for biosimilars. Moreover, regulators should collaboratively strive for a unified, scientific EU position on the interchangeability of biosimilars.

## Data Availability Statement

The original contributions presented in the study are included in the article/Supplementary Material, with exception of the interview datasets. These are not readily available because they contain information that could compromise interviewees' privacy and consent. Further inquiries can be directed to the corresponding author.

## Ethics Statement

Ethics approval for the interviews was granted by the Research Ethics Committee UZ/KU Leuven (MP007272, Belgium).

## Author Contributions

LB, IH, AV, PD, and SS were involved in the development of the study design. LB and AM were involved in data collection and analysis. LB wrote the first draft of the manuscript. All authors reviewed the draft manuscript, added suggestions, and read and approved the final manuscript.

## Funding

This work was supported by KU Leuven and the KU Leuven Fund on Market Analysis of Biologics and Biosimilars following Loss of Exclusivity (MABEL Fund).

## Conflict of Interest

IH, SS, PD, and AV are the founders of the KU Leuven Fund on Market Analysis of Biologics and Biosimilars following Loss of Exclusivity (MABEL Fund). AV is involved in consulting, educational work and speaking engagements for a number of companies, i.e., AbbVie, Accord, Amgen, Biogen, Effik, EGA, Pfizer/Hospira, Fresenius-Kabi, Mundipharma, Roche, Novartis, Sandoz, Boehringer Ingelheim. SS was involved in a stakeholder roundtable on biologics and biosimilars sponsored by Amgen, Pfizer, and MSD, and he has participated in advisory board meetings for Amgen, Pfizer, and Sandoz. He has contributed to studies on biologics and biosimilars for Hospira, Celltrion, Mundipharma, and Pfizer; and he has had speaking engagements for Amgen, Celltrion and Sandoz. PD participated at advisory board meetings for AbbVie, Amgen, Hospira, and Samsung Bioepis and is on the Speakers' Bureau of AbbVie, Celltrion, Hospira, Merck Serono, and Roche. The remaining authors declare that the research was conducted in the absence of any commercial or financial relationships that could be construed as a potential conflict of interest.

## Publisher's Note

All claims expressed in this article are solely those of the authors and do not necessarily represent those of their affiliated organizations, or those of the publisher, the editors and the reviewers. Any product that may be evaluated in this article, or claim that may be made by its manufacturer, is not guaranteed or endorsed by the publisher.
