# Supplementary material for: Regulatory Information and Guidance on Biosimilars and Their Use Across Europe: A Call for Strengthened One Voice Messaging
Source: Front Med (Lausanne). 2022 Mar 9;9:820755. doi: 10.3389/fmed.2022.820755 (PMC8959407; doi:10.3389/fmed.2022.820755)
Supplement: Supplementary file 1 [file Data_Sheet_1.docx]

Supplementary Material

Regulatory Information and Guidance on Biosimilars and their Use across Europe: a call for strengthened one voice messaging

Liese Barbier^1*^, Allary Mbuaki^1^, Steven Simoens^1^, Paul Declerck^1^, Arnold G. Vulto^1, 2^, Isabelle Huys^1^

^1^KU Leuven, Department of Pharmaceutical and Pharmacological Sciences, Herestraat 49 box 521, 3000 Leuven, Belgium

^2^Hospital Pharmacy, Erasmus University Medical Center, box 2040, 3000 CA Rotterdam, the Netherlands

*** Correspondence:**Liese Barbier
liese.barbier@kuleuven.be

- **Table S1.** List of national competent authorities in the EEA + UK (N countries=31)*
- **Table S2.** Overview of members of the Biosimilar Medicinal Products Working Party
- **Table S3.** Overview of topics discussed during the semi-structured interviews (qualitative study phase)
- **Table S4.** Type of educational material on the NCA website about biosimilars (available from 16 of 31 national medicines agencies)
- **Table S5.** Positions from national medicines agencies about the interchangeability of biosimilars with their reference in Europe: guidance provided by 8 of 31 agencies
- **Table S6.** Positions from national medicines agencies about switching between reference biological medicines and biosimilars in Europe: guidance provided by 12 of 31 agencies
- **Table S7.** Positions by national medicines agencies about (automatic) substitution for biological medicines: guidance provided by 10 of 31 agencies
- **Table S8.** Interview participants’ characteristics
- **Table S9.** Interview participants’ characteristics
- **Box S1.** Interchangeability: different regulatory interpretations and implications in different regions across the world
- **Figure S1.** Overview of rapporteurship for centrally evaluated biosimilar over time

| **Table S1. List of national competent authorities in the EEA**(1) **+ UK (N countries=31)*** | | | |
| --- | --- | --- | --- |
| **N** | **Country** | **Name NCA** | **Link to website** |
|  | Austria | - Austrian Agency for Health and Food Safety (AGES) - Austrian Federal Office for Safety in Health Care (BASG) | - [www.ages.at](http://www.ages.at) - <https://www.basg.gv.at/> |
|  | Belgium | - Federal Agency for Medicines and Health Products (FAMHP) | - [www.fagg-afmps.be/](http://www.fagg-afmps.be/) |
|  | Bulgaria | - Bulgarian Drug Agency | - [www.bda.bg](http://www.bda.bg) |
|  | Croatia | - Agency for medicinal products and medical devices of Croatia | - [www.halmed.hr](http://www.halmed.hr) |
|  | Cyprus | - Ministry of Health - Pharmaceutical Services | - [www.moh.gov.cy/phs](http://www.moh.gov.cy/phs) |
|  | Czechia | - State Institute for Drug Control | - [www.sukl.cz](http://www.sukl.cz) |
|  | Denmark | - Danish Medicines Agency (Laegemiddelstyrelsen) | - [www.laegemiddelstyrelsen.dk](http://www.laegemiddelstyrelsen.dk) |
|  | Estonia | - State Agency of Medicines | - [www.ravimiamet.ee](http://www.ravimiamet.ee) |
|  | Finland | - Finnish Medicines Agency (FIMEA) | - [www.fimea.fi](http://www.fimea.fi) |
|  | France | - National Agency for the Safety of Medicine and Health Products (ANSM) | - [www.ansm.sante.fr](http://www.ansm.sante.fr) |
|  | Germany | - Federal Institute for Drugs and Medical Devices - Paul Ehrlich Institute | - [www.bfarm.de](http://www.bfarm.de) - [www.pei.de/](http://www.pei.de/) |
|  | Greece | - National Organization for Medicines | - [www.eof.gr](http://www.eof.gr) |
|  | Hungary | - National Institute of Pharmacy and Nutrition | - [www.ogyei.gov.hu](http://www.ogyei.gov.hu) |
|  | Iceland | - Icelandic Medicines Agency | - [www.ima.is](http://www.ima.is) |
|  | Ireland | - Health Products Regulatory Authority (HPRA) | - [www.hpra.ie](http://www.hpra.ie) |
|  | Italy | - Italian Medicines Agency | - [www.aifa.gov.it](http://www.aifa.gov.it) |
|  | Latvia | - State Agency of Medicines | - [www.zva.gov.lv](http://www.zva.gov.lv) |
|  | Liechtenstein | - Office of Health / Department of Pharmaceuticals | - <https://www.llv.li> |
|  | Lithuania | - State Medicines Control Agency | - [www.vvkt.lt](http://www.vvkt.lt) |
|  | Luxembourg | - Ministry of Health | - [www.ms.etat.lu](http://www.ms.etat.lu) |
|  | Malta | - Medicines Authority | - [www.medicinesauthority.gov.mt](http://www.medicinesauthority.gov.mt) |
|  | Netherlands | - Medicines Evaluation Board | - <https://www.cbg-meb.nl/> |
|  | Norway | - Norwegian Medicines Agency | - <https://legemiddelverket.no> |
|  | Poland | - Office for Registration of Medicinal Products, Medical Devices and Biocidal Products - Chief Pharmaceutical Inspectorate | - [www.urpl.gov.pl](http://www.urpl.gov.pl) - [www.gif.gov.pl](http://www.gif.gov.pl) |
|  | Portugal | - National Authority of Medicines and Health Products | - [www.infarmed.pt](http://www.infarmed.pt) |
|  | Romania | - National Authority of Medicines and Medical Devices | - [www.anm.ro](http://www.anm.ro) |
|  | Slovakia | - State Institute for Drug Control | - [www.sukl.sk](http://www.sukl.sk) |
|  | Slovenia | - Agency for Medicinal Products and Medical Devices of the Republic of Slovenia | - [www.jazmp.si](http://www.jazmp.si) |
|  | Spain | - Spanish Agency for Medicines and Health Products | - [www.aemps.gob.es](http://www.aemps.gob.es) |
|  | Sweden | - Medical Products Agency | - [www.lakemedelsverket.se](http://www.lakemedelsverket.se) |
|  | UK | - Medicines and Healthcare Products Regulatory Agency - National Health Service (NHS) | - <https://www.gov.uk/> - <https://www.england.nhs.uk/medicines/> |
| ** Some countries have multiple agencies/organizations, with each an own website. Information was merged and only counted once.* | | | |

| Table S2. Overview of members of the Biosimilar Medicinal Products Working Party (2) (composition 2021) | | |
| --- | --- | --- |
| Represented national medicines agency | **Country** | **Function** |
| PEI | Germany | Chair |
| FIMEA | Finland | Vice-Chair |
| AGES | Austria | Member |
| FAMHP | Belgium | Member |
| Danish Medicines Agency | Denmark | Member |
| ANSM | France | Member |
| BfArM | Germany | Member |
| HPRA | Ireland | Member |
| MEB | Netherlands | Member |
| MPA | Sweden | Member |

| Table S3. Overview of topics discussed during the semi-structured interviews (qualitative study phase) |
| --- |
| During the interviews participants were asked about |
| 1. Their perspective on the regulatory guidance and educational informational about biosimilars provided by regulatory authorities, both by the European Medicines Agency and national competent authorities. Questions were posed on the type of information which is made available, the way in which regulators disseminate information, the role of the regulatory agencies on central and national level in informing, educating and guiding stakeholders, the role of other stakeholder organizations, the type of information deemed important and the role of the European Public Assessment Report 2. Their preferences, needs and suggestions on how to guide and inform their stakeholder group about the evaluation and use of biosimilars. Questions were posed about the guidance provided regarding biosimilar interchangeability, switching and substitution. |

| **Table S4. Type of educational material on the NCA website about biosimilars (available from 16 of 31 national medicines agencies)** | | |
| --- | --- | --- |
| **Country** | **Available educational materials & educational activities** | **EMA/EC material provided*** |
| Belgium | - Multi-stakeholder symposium “Biological medicines in Belgium” with presentations available on website (February 2018) - Information campaign aimed at informing HCPs and patients about biological including biosimilar medicines. The campaign included a dedicated website, brochures, posters, and radio spots (launched in December 2018) | / |
| Croatia | / | Patient information guide and HCP information guide (in English and Croatian) |
| Denmark | - Videos about original and biosimilar biological medicines and switching to a biosimilar medicinal product - Booklet for patients, flyer for health professionals - Text proposal for hospital intranet products - The listed information materials were prepared as part of an Action Plan on Biological Medicines, Biosimilar Drugs and Vaccines 2015 - 2016 | / |
| Finland | - Video on biosimilars: “Biosimilaari on varteenotettava vaihtoehto” (September 2017) - Slide show for health care organizations on the importance to deploy biosimilar (March 2017) | / |
| France | - ANSM report: “État des lieux sur les medicaments biosimilaires” (May 2016) - Expert report from the AFSSAPS: “Des médicaments issus des biotechnologies aux médicaments biosimilaires: état des lieux” (July 2011) | / |
| Germany | - Dialogue event about biosimilars “BfArM im Dialog: Biosimilars”. Presentations of speakers available on the website (June 2016) - DRGA - Workshop Biosimilars financed by the German Statutory Health Insurance System (GKV) | / |
| Hungary | - Physical-pharmaceutical information days: trends and innovations in drug therapy (October 2017) | Information guide for HCPs (in Hungarian) |
| Iceland | / | Information guide for HCPs (in English) |
| Ireland | - HPRA Guide for Heath care professionals and patients - HPRA video: “Biosimilars explained – HPRA” - HPRA’s information document for patients : " Biological and biosimilar medicines: What patients should know" (August 2017) - Information evening for healthcare professionals on biosimilar medicines (October 2017). Presentations available on website | / |
| Italy | - Position Paper on biosimilars (March 2018) | Video (in Italian) |
| The Netherlands | - Extra medical information for healthcare providers: differences between biological medicines and biosimilars (webpage) - Answers to questions about biological medicines (for patients and consumers), developed with input from patient organisations (April 2018) - Presentation "What are biosimilars" | Link to information guide for HCPs |
| Portugal | - Patient information document on Biosimilar medications from Infarmed | Patient information guide (in Portuguese) |
| Slovakia | / | Patient information guide (in Slovak) |
| Spain | / | Patient information guide (in Spanish) |
| Sweden | - Manufacturer-independent information on biosimilars by TLV (2017) | / |
| UK | - NHS England information guide "What is a biosimilar medicine?", a non-prescriptive consensus document that provides key information on biosimilar medicines. (September 2015) | / |
| **EMA/EC’s HCP and/or patient guide and/or animated video about biosimilar medicines*  *AFSSAPS: Agence Française de Sécurité Sanitaire des Produits de Santé, ANSM: Agence nationale de sécurité du médicament et des produits de santé, EC: European Commission, EMA: European Medicines Agency, HPRA: Health Products Regulatory Authority, NCA: national competent authority, NCA: national competent authority, NHS: national health service, Q&A: question and answer, TLV: Swedish Dental and Pharmaceutical Benefits Agency*  *Some relevant updates since end of data collection (March 2019):*   - *UK: NHS England information guide was updated and republished in May 2019* - *Ireland: HPRA information guide was updated with comments collected during a public consultation and republished in August 2020* - *Finland: Links to the EMA patient Q&A and HCP information guide are now available on FIMEA’s website. FIMEA affiliated researchers conducted a study on physician views about biosimilars, and substitution for biologicals, with accompanying information documents explaining results (May 2019).* | | |

| **Table S5. Positions from national medicines agencies about the interchangeability of biosimilars with their reference in Europe: guidance provided by 8 of 31 agencies** | |
| --- | --- |
| **NCA’s indicating that reference and biosimilar products can be used interchangeably** | |
| Croatia | “A biosimilar medicinal product and originator biological medicinal product can be used interchangeably in the treatment of patients, under medical surveillance and monitoring of the patient's health condition.” (2019) |
| Finland | “The current position of Fimea is that biosimilars are interchangeable with their reference products under the supervision of a health care person.” (2015) |
| France | “Interchangeability is a medical procedure that consists, at the initiative of the prescriber, to replace a biological drug with a similar one. It can occur at any time during treatment. It must be reasoned and take into account the interest of the patient. The following three conditions must be met: inform the patient and obtain his agreement; provide appropriate clinical monitoring during treatment; ensure traceability on the products concerned (the prescribed product must be entered in the patient's file).”* |
| Italy | “As demonstrated by the regulatory authorization process, the risk-benefit ratio of biosimilars is the same as that of the reference originators. For this reason, AIFA considers biosimilars as interchangeable products with the corresponding reference originators. This consideration is valid both for naïve patients and for patients already under treatment.”* (2018) |
| The Netherlands | “The exchange between an original biological medicines and a biosimilar is possible, but needs to be done under supervision of the physician and in consultation with the patient. Biosimilars can also be interchanged, but only if they are based on the same original biological medicine”* (2015) |
| **NCA’s indicating that interchangeable use of reference and biosimilar products is an ongoing area of debate** | |
| Ireland | “Interchangeability between biosimilars and reference products is an ongoing area of debate. While prescribing practices are at the discretion of healthcare professionals, the HPRA does not recommend that patients are switched back and forth between a biosimilar and the reference medicinal product.” |
| UK | “The evidence regarding interchangeability is still developing.” “Generally allowed under physician's supervision.” |
| **NCA’s indicating that reference and biosimilar products cannot be used interchangeably** | |
| Sweden | “The Medical Products Agency's assessment based on current scientific evidence is that biological drugs are not interchangeable at pharmacy level. The assessment is mainly based on the fact that the risk of immunological reactions during frequent changes is incompletely elucidated.”* |
| *AIFA: L'Agenzia Italiana del Farmaco, Fimea: Finnish Medicines Agency, HPRA: Health Products Regulatory Authority, NCA: national competent authority *Translation in English from a national (non-English) source text* | |

| **Table S6. Positions from national medicines agencies about switching between reference biological medicines and biosimilars in Europe: guidance provided by 12 of 31 agencies** | |
| --- | --- |
| **NCAs endorsing the practice of switching between a reference biological and biosimilar** | |
| Belgium | “If the prescriber decides to move from one to the other (original/original; original/biosimilar; biosimilar/original or biosimilar/biosimilar, often also called "switch" in this context), then this must be done with the necessary follow-up and the modification must be recorded accurately. The exclusion of INN prescription avoids switching without follow-up by the prescriber. However, since the biosimilar medicinal product can only be authorised if it has the same safety and efficacy profile as the reference medicinal product, relevant changes in treatment are not expected upon switching from the reference product to a biosimilar medicinal product (or vice versa)” |
| Denmark | “Would it be problematic to switch to a biosimilar medicinal product? No. The biosimilar medicinal product can only be authorised if it has the same efficacy profile as the reference medicinal product, and consequently you will not experience any changes in your treatment if you switch to a biosimilar medicinal product.” “If you are in treatment with an original biologic medicine and switch to a biosimilar medicine, you will not experience any difference in the effect of treatment.” |
| Finland | “Switches between biological products are common and usually not problematic, e.g. in the context of hospital tendering processes, for time being, there is no evidence for adverse effects due to the switch from a reference product to a biosimilar, the theoretical basis of such adverse effects is weak.” |
| Germany | “So far, the Paul-EhrIich-Institut has not received any report that switching from any reference product to a biosimilar product has led to problems in the treatment of patients. An increasing number of publications in the scientific literature can be found on biosimilars indicating that no safety problems occur when switching from an originator product to a biosimilar.” |
| Italy | “Numerous analyses published in the literature have compared the effects on patients of the transition from the original biological drugs to their biosimilars (the so-called "switch"), highlighting completely similar outcomes, ...”* |
| Norway | “The position of the Norwegian Medicines Agency is that switching between reference products and biosimilars during ongoing treatment, is safe. It can apply to the following situations:  1) Switching from reference drug to biosimilar. 2) Switching from biosimilar to reference drug. 3) Switching from a biosimilar to another biosimilar based on the same reference product. The decision on switching products is made by the treating physician or hospital, who have to provide the necessary information to patients. All patients treated with biological drugs must receive the necessary follow-up. To ensure traceability, adverse reactions with biological drugs should be reported with the drug name, active substance and batch number.” “Switching is necessary to achieve competition between equally efficient drugs. Competition leads to price reductions that reduce the financial burden of expensive biological drugs in the healthcare system.” |
| Portugal | “Similarly, in the case of patients already undergoing treatment there is evidence to suggest that switching from a reference biological drug to a biosimilar drug can be effected without loss of efficacy or increased risk of adverse reactions.” “All the prescribers and other technicians involved in the switching program (physicians, pharmacists and nurses) must be involved and informed about the process and their benefits. Also in this case, the guidance foresees that this decision is taken by the prescriber in an informed procedure, in an informed manner, by the patient.” “As indicated in the guidance, there is no obligation to change treatment for a more cost-effective alternative. However, if you choose not to do so, you must justify your decision to the local CFT.” “When the assessment of alternative brands of the same biologic medicine translates in a significant reduction of costs in patients already under treatment with a biological medicinal product, a process of switch for the lowest cost medicine should be implemented.”* |
| The Netherlands | “Switching to another biological medication is done in consultation with the patient, doctor and (hospital) pharmacist. Your doctor and the (hospital) pharmacist will keep an eye on whether the medicine is working and whether any side effects occur.” “Exchange between biological medicines (regardless of whether this concerns innovator products or biosimilar medicines) is possible, however only if adequate clinical monitoring is performed and the patient is well informed.””* |
| **NCAs stating that it is up to the discretion of the prescriber, without providing further guidance** | |
| Croatia | “The appropriateness of switch for the individual patient should be decided by the prescribing doctor after having taken in consideration all the relevant above-mentioned facts.” |
| UK | “Guidance across some EU Member States currently recommends that switching between a reference product and its biosimilar (and indeed amongst biosimilar medicines) should be managed at the discretion of the individual prescriber in partnership with the patient, with appropriate monitoring in place” |
| **NCAs discouraging repeated switching** | |
| Ireland | ““If it is planned to change the medicine a patient receives from a reference to a biosimilar medicine or vice versa, the treating physician should be involved; this should involve discussion between the prescriber/ patient, and prescriber/dispensing pharmacist.” “The HPRA does not recommend that patients are switched back and forth between a biosimilar and the reference medicinal product. It is recommended that there is consultation between prescribers, pharmacists and procurement staff in relation to deciding on treatment preferences for using a reference or a biosimilar medicine.” |
| Sweden | “The choice between reference medicine and biosimilar when starting treatment as well as any altered prescription during ongoing treatment (switch) between the reference product and the biosimilar is done in Sweden by prescribing physicians in consultation with the patient. For the time being, repeated changes between biosimilar and reference product (multiple switch) are not recommended, an assessment primarily based on the fact that the risk of immunological reactions during frequent changes is incompletely elucidated. Additional clinical experience and relevant studies are needed to bring clarity in this area.”* |
| *EU: European Union, HPRA: Health Products Regulatory Authority, NCA: national competent authority*  **Translation in English from a national (non-English) source text* | |

| **Table S7. Positions by national medicines agencies about (automatic) substitution for biological medicines: guidance provided by 10 of 31 agencies** | |
| --- | --- |
| **(Automatic) substitution for biologicals is not allowed** | |
| Belgium | “Substitution (the passage of a specialty subject to a prescription to another specialty by the pharmacist, without consulting the doctor) is not allowed in Belgium for biologicals (including biosimilars).” |
| Croatia | “The direct interchangeability (known also as direct, automatic or generic substitution) is a notion that includes a substitution of the prescribed medicinal product with its parallel at the level of pharmacy, which does not require advising and special surveillance of the prescribing doctor. The direct interchangeability is only possible for generic medicinal products, whereas for biological medicinal products, direct interchangeability is not applicable.” |
| Portugal | “Automatic substitution by the pharmacy is not allowed. In case of refusal of the change, the hospital pharmacy will make available the medicine that the patient already used.”* |
| Finland | “The pharmacovigilance view is that the biosimilar contains a new version of the active ingredient of the original drug, the similarity of which has been demonstrated in extensive experiments. The biosimilar is therefore, in principle, interchangeable. Interchangeability should be assessed on a case-by-case basis for each biosimilar, as the exchange may involve specific issues related to the medicinal product, the drug delivery device and the patient. At least for the time being, a drug exchange at a pharmacy is not possible between biosimilar and original drugs.”* |
| Ireland | “This will also ensure that substitution of biosimilar medicines does not inadvertently occur when the medicine is dispensed by the pharmacist.” “Under the Health (Pricing and Supply of Medical Goods) Act 2013, the HPRA publishes a list of medicines which may be substituted for each other in the community pharmacy setting. When generic medicines are made available to patients in this way, it is known as ‘generic substitution’. It is important to highlight that under this legislation biological medicines are specifically excluded from being added to interchangeable medicine lists. As such, they cannot be subjected to pharmacy substitution.” |
| Netherlands | “Exchange between biological medicines (regardless of whether this concerns innovator products or biosimilar medicines) is possible, however only if adequate clinical monitoring is performed and the patient is well informed.” “Avoid uncontrolled exchange between biological medicines (irrespective of whether they are reference medicines or biosimilars). This means that a patient is adequately monitored clinically and receives clear instructions.”* |
| Sweden | “Interchangeability at pharmacies is decided by the Medical Products Agency. The Medical Products Agency's assessment based on current scientific evidence is that biological drugs are not interchangeable at pharmacy level. The assessment is mainly based on the fact that the risk of immunological reactions during frequent changes is incompletely elucidated.”* |
| UK | “When prescribing biological products, it is good practice to use the brand name.” (info from GO.UK)  “This will ensure that automatic substitution of a biosimilar product does not occur when the medicine is dispensed by the pharmacist.” (info from NHS guide on biosimilars) |
| **(Planned) changes to legislation to allow substitution for (certain) biologicals** | |
| Germany | The extent to which a physician is consulted before a decision is taken on which of the medicinal products with comparable therapeutic risk/benefit ratio shall be administered (originator product or biosimilar) – in other words, whether or not a product is automatically substituted – is not within the competence of the national or European regulatory authority but rather depends on the respective national health care systems. (PEI) |
| Norway | In Norway, automatic substitution in pharmacies of biological or biosimilar products is not allowed. The Norwegian Medicines Agency has proposed that the Pharmacy Act § 6-6, which is the basis for generic (automatic) substitution in pharmacies, should be altered, eventually permitting automatic substitution of new classes of medicinal products, e.g. biological drugs |
| **Translation in English from a national (non-English) source text*  *Germany New legislation planned (GSAV: Gesetz für mehr Sicherheit in der Arzneimittelversor-gung), that will allow biologicals to be substituted at pharmacy level* | |

| Table S8. Interview participants’ characteristics | | | |
| --- | --- | --- | --- |
| Stakeholder | **N=14** | **Therapeutic area/**  **business area** | **Company type** |
| Healthcare professionals | 7 | Oncologist (2)  Rheumatologist (2)  Gastro-enterologist (1)  Pharmacist specialized in biosimilar policy (1)  Physician with specialty in EU regulatory framework (1) | / |
| Pharmaceutical industry | 7 | Regulatory affairs (2)  Medical Affairs (3)  Public Affairs (2) | Pharmaceutical industry umbrella organization (3)  Legacy originator company (2)  Legacy biosimilar company (2) |

| Table S9. Interview participants’ characteristics | | | | |
| --- | --- | --- | --- | --- |
|  | **Interview identifier** | **Stakeholder group** | **Therapeutic area/**  **pharmaceutical industry expertise** | **Pharmaceutical industry company type** |
| 1 | HCP1 | Healthcare professional | Physician with specialty in EU regulatory framework | / |
| 2 | HCP2 | Healthcare professional | Oncologist and medical association representative | / |
| 3 | HCP3 | Healthcare professional | Rheumatologist and medical association representative | / |
| 4 | HCP4 | Healthcare professional | Rheumatologist | / |
| 5 | HCP5 | Healthcare professional | Gastroenterology and medical association representative | / |
| 6 | HCP6 | Healthcare professional | Oncologist and medical association representative | / |
| 7 | HCP7 | Healthcare professional | Pharmacist specialized in biosimilar policy | / |
| 8 | I1 | Pharmaceutical industry | Regulatory affairs | Legacy originator company |
| 9 | I2 | Pharmaceutical industry | Public affairs | Pharmaceutical industry umbrella organization |
| 10 | I3 | Pharmaceutical industry | Medical affairs | Legacy generic company |
| 11 | I4 | Pharmaceutical industry | Medical affairs | Legacy originator company |
| 12 | I5 | Pharmaceutical industry | Medical affairs | Legacy generic company |
| 13 | I6 | Pharmaceutical industry | Regulatory affairs | Pharmaceutical industry umbrella organization |
| 14 | I7 | Pharmaceutical industry | Public affairs | Pharmaceutical industry umbrella organization |

| **Box S1. Interchangeability: different regulatory interpretations and implications in different regions across the world** | |
| --- | --- |
| **United States** | - The US Food and Drug Administration (FDA) has a dedicated separate regulatory pathway for biosimilar interchangeability designation (3),(4). As set out in the Biologics Price Competition and Innovation Act (BPCIA) of 2009, the FDA has the authority to designate biologicals as interchangeable.(5) According to the BPCIA, interchangeable biologics are biosimilars that “*can be expected to produce the same clinical result as the reference product in any given patient”*. Interchangeability designation allows pharmacists to substitute a biosimilar for its reference product without the intervention of the prescriber, if consistent with state laws (6). In the US, interchangeability is thus a formal designation that regulates pharmacist substitution practices. - To receive interchangeability designation, a biosimilar product needs to meet a specific set of additional requirements beyond these for biosimilarity demonstration (7). In May 2019, the FDA finalized its guidance for industry on interchangeability demonstration, which outlines the requirements to demonstrate interchangeability with a reference biological, including the need for clinical data from one or more alternating switch studies in most instances (5),(8). Recently, a first biosimilar has received interchangeability designation, i.e. Semglee®, an insulin glargine biosimilar (8). |
| **Australia** | - Interchangeability is not part of regulatory biosimilar evaluation by the Australian medicine and therapeutic regulatory agency, Therapeutics Goods Administration (TGA), and is in that regard similar to Europe. - The pathway to allow for pharmacist led substitution is coordinated by the Pharmaceutical Benefits Advisory Committee (PBAC) as part of the payer’s decision. The PBAC can recommend biosimilars as equivalents with their reference product and other biosimilars of the same reference product, allowing them to be substituted by a pharmacist (9). Biosimilars deemed substitutable at pharmacy level receive a so called ‘a-flag’. PBAC recommendations regarding substitution are made on a case-by-case basis, and may take into account the availability of supportive data regarding the effect of switching between reference and biosimilar products, and practical elements that could affect easiness of substitution by the pharmacist at the point of dispensing. The latter may include for example the strength of formulation and the number of units per product pack (9). Several biosimilars have received such ‘a-flag’ and are therefore suitable for substitution at the pharmacy. |

**Figure S1 Overview of rapporteurship for centrally evaluated biosimilar over time**

N biosimilars (2006-2020, excluding duplicates) = 51

*Biosimilars that received marketing authorization or received a position opinion and were pending EC decision between 2006 and 2020 were considered. Product that were withdrawn post-authorization were excluded. Duplicates were excluded.

**References**

1. European Medicines Agency. National competent authorities (human) [Internet]. [cited 2021 Feb 17]. Available from: https://www.ema.europa.eu/en/partners-networks/eu-partners/eu-member-states/national-competent-authorities-human

2. European Medicines Agency. Biosimilar Medicinal Products Working Party [Internet]. [cited 2021 May 25]. Available from: https://www.ema.europa.eu/en/committees/working-parties-other-groups/chmp/biosimilar-medicinal-products-working-party

3. Ebbers HC, Schellekens H. Are we ready to close the discussion on the interchangeability of biosimilars? Drug Discov Today. 2019;24(10):1963–7.

4. O’Callaghan J, Barry SP, Bermingham M, Morris JM, Griffin BT. Regulation of biosimilar medicines and current perspectives on interchangeability and policy. Eur J Clin Pharmacol. 2019;75(1):1–11.

5. U.S. Food & Drug Administration. Considerations in Demonstrating Interchangeability With a Reference Product Guidance for Industry Considerations in Demonstrating Interchangeability With a Reference Product Guidance for Industry. 2019.

6. CardinalHealth. State laws for biosimilar interchangeability [Internet]. [cited 2021 Aug 28]. Available from: https://www.cardinalhealth.com/en/product-solutions/pharmaceutical-products/biosimilars/state-regulations-for-biosimilar.html?cid=GEN-VURL-PHR-CHSS-BSM-State_laws-Biosimilars

7. U.S. Food & Drug Administration. Biosimilar and Interchangeable Products [Internet]. [cited 2021 May 18]. Available from: https://www.fda.gov/drugs/biosimilars/biosimilar-and-interchangeable-products

8. U.S. Department of Health and Human Services F and DA, (CDER) C for DE and R. Clinical Immunogenicity Considerations for Biosimilar and Interchangeable Insulin Products. FDA Guidance for Industry. 2019.

9. Australian Government Department of Health. Who chooses whether the biosimilar medicine or the reference biological medicine is used? [Internet]. [cited 2021 May 18]. Available from: https://www1.health.gov.au/internet/main/publishing.nsf/Content/biosimilar-hp-who-chooses-whether-biosimilar-medicine-or-reference-biological-medicine-is-used
